# Molecular data on the CO1 and beta fibrinogen gene in the evolutionary relationships of the mastiff bat (Chiroptera, Molossidae, *Molossus*)

**DOI:** 10.1016/j.dib.2018.04.088

**Published:** 2018-04-30

**Authors:** Livia O. Loureiro, Burton K. Lim, Mark D. Engstrom

**Affiliations:** a Department of Ecology and Evolution, University of Toronto, Toronto, ON, Canada M5S 3B2; b Department of Natural History, Royal Ontario Museum, Toronto, ON, Canada M5S 2C6

**Keywords:** Molossidae, Phylogenetics, Neotropics, Evolution

## Abstract

*Molossus* is one of the most diverse genera of free-tailed bats in the pantropical family Molossidae and occurs though all the Neotropics. Nevertheless, the taxonomy and phylogeny of this group is poorly understood. Here, we present the data on evolutionary relationships of *Molossus* based on DNA barcodes of COI gene from 346 specimens of *Molossus* and its sister genus *Promops* and another New World molossid *Eumops*. Of these specimens, 50 are new sequences and 296 were obtained from GenBank. In addition, the nuclear gene beta fibrinogen was sequenced from a subset of 35 specimens. These data provide the basis for further exploration and understanding of the phylogenetic relationships of the genus *Molossus* (Loureiro et al., 2018) [1].

**Specifications table**

**Table:** 

Subject area	*Biology*
More specific subject area	*Genetics, Molecular phylogeny*
Type of data	*Figures*
How data was acquired	*Tissues were extracted using the DNeasy Tissue Kit (QIAGEN Inc., Valencia, California). PCR protocols followed*[2], [3], [4]. *The nucleotides were sequenced in an ABI 3130* (*Applied Biosystems_*) *automated sequencer using Big Dye Terminator Cycle Sequencing methodology* (*Applied Biosystems_*)*.*
Data format	*Analyzed*
Experimental factors	*Total genomic DNA was extracted from liver, heart or kidney tissue that were frozen in liquid nitrogen or preserved in ethanol.*
Experimental features	*Sequences were assembled in SEQUENCHER and aligned using the Muscle algorithm*[5]. Phylogenetic relationships were reconstructed MEGA 6.06 [6].
Data source location	Bonaire, Dominican Republic, Ecuador, El Salvador, French Guiana, Guyana, Jamaica, Martinique, Mexico, Panama, Peru, Suriname, Venezuela, Bolivia, and Montserrat.
Data accessibility	*The sequences have been deposited in the public repository of GenBank and BOLD systems* (Supplementary material 1)

**Value of the data**

• Phylogenetic relationships within the genus remain undefined and until recently there have been few molecular studies of *Molossus* [1], [11], [12]. Therefore, these data combined with more genetic markers, more species of the genus, and more comprehensive geographic sampling could clarify the evolution of *Molossus*.
• These data could help to test the homology of many morphological and ecological characters, such as echolocation calls.
• *Molossus* is a Neotropical genus, occurring from the southeastern United States to southern Argentina, and throughout the Caribbean islands. Therefore, these data could be used in the development of biogeographic studies in the Neotropics.
• This data could be used for comparative studies related to other genera of molossid bats to understand the evolution of the family Molossidae.

## 1 Data

**Fig. 1:**
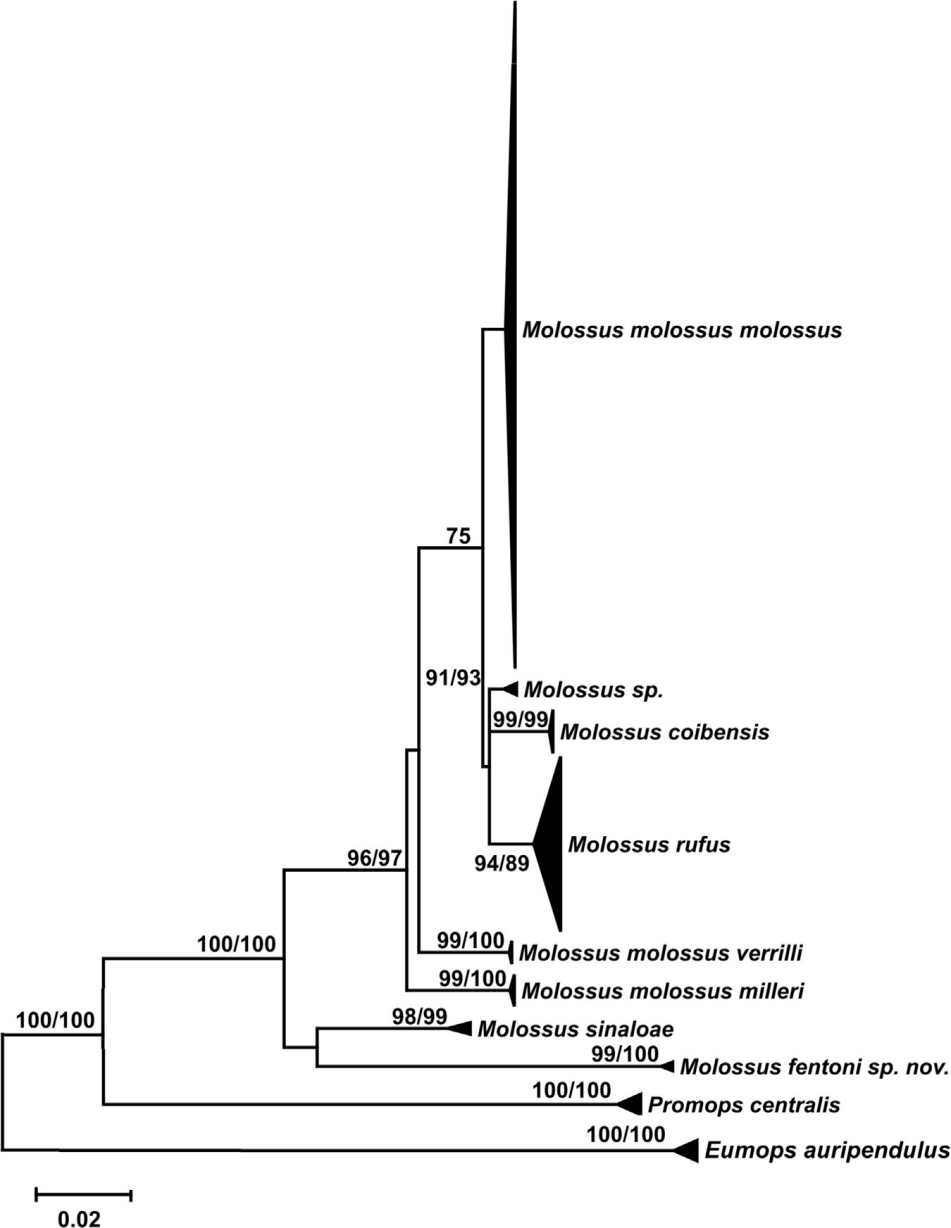
Maximum likelihood tree of COI sequences of *Molossus*. Bootstrap support values (maximum likelihood/maximum parsimony) >70% are reported for well-supported nodes. *M. m. daulensis* was recovered inside the *M. m. molossus* clade.

**Fig. 2:**
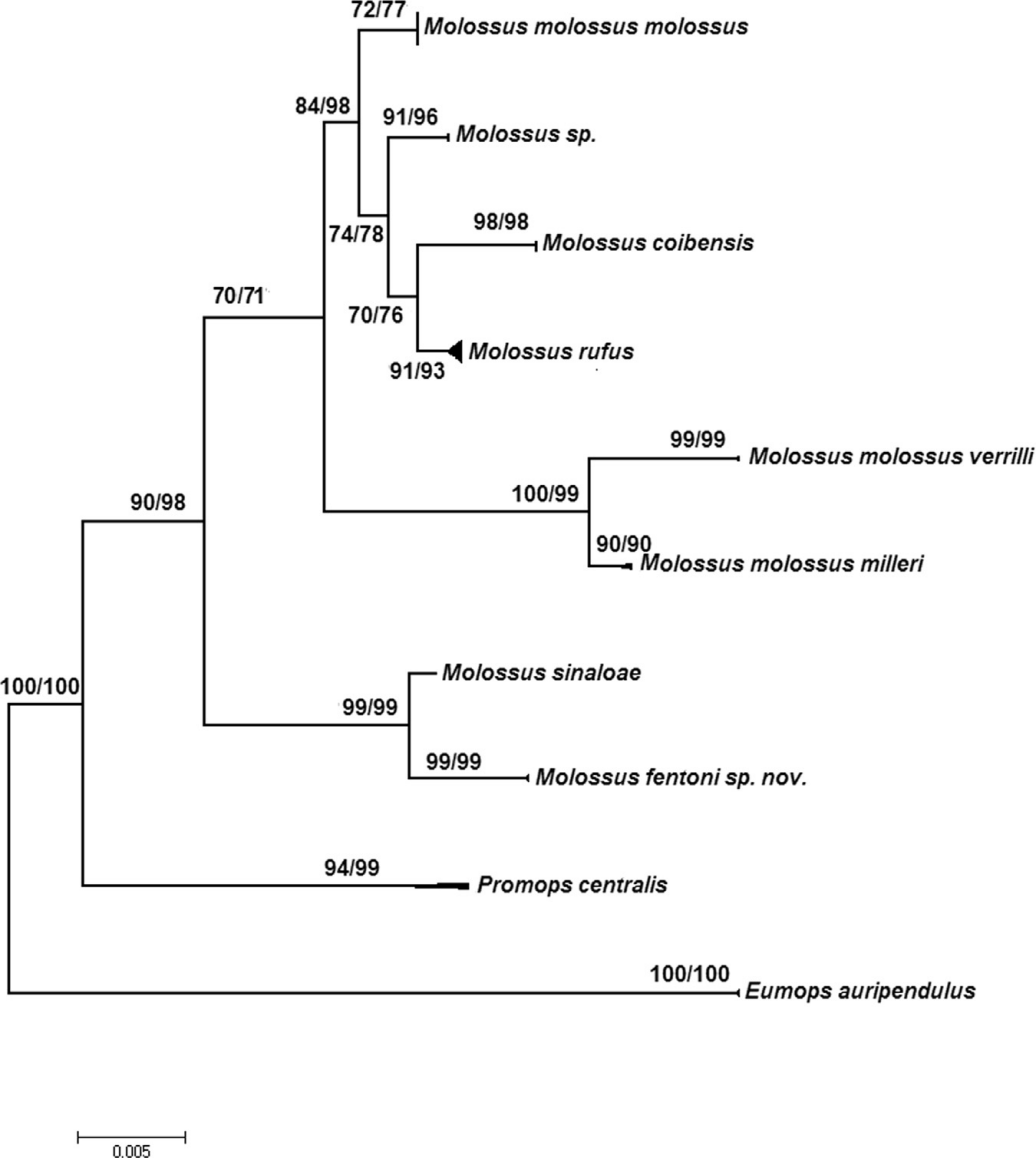
Maximum likelihood tree of beta-fibrinogen sequences of *Molossus*. Bootstrap support values (maximum likelihood/maximum parsimony) >70% are reported for well-supported nodes. *M. m*.

*Molossus* is one of the most diverse and common genera of the family Molossidae, but its taxonomy and phylogenetic relationships are still poorly understood. Maximum likelihood trees for the mitochondrial CO1 gene (Fig. 1) and nuclear beta fibrinogen gene (Fig. 2) from more than 300 specimens of *Molossus* and it sisters groups distributed through all the Neotropics estimate the phylogenetic relationships within the genus. We also present the specimen vouchers used in the genetic analyses for the genes COI and Beta fibrinogen, the GenBank and BOLD Systems accessions numbers, the species identification, and country where the specimens were collected (Supplementary material 1).

## 2 Experimental design, materials and methods

### 2.1 DNA sequencing

Total genomic DNA was extracted from liver, heart or kidney tissue that were frozen in liquid nitrogen or preserved in ethanol. Tissues were extracted using the DNeasy Tissue Kit (QIAGEN Inc., Valencia, California) following the manufacturer's protocol. Molecular protocols for the COI gene followed the methods outlined by Refs. [2], [3] and protocols for the beta fibrinogen gene followed [4]. The nucleotides of both strands were sequenced in an ABI 3130 (Applied Biosystems_) automated sequencer using Big Dye Terminator Cycle Sequencing methodology (Applied Biosystems_). These analyses were carried out in accordance with the recommendations of the Canadian Council on Animal Care, the requirements under the Animals for Research Act (Revised Statutes of Ontario, 1990), and the Royal Ontario Museum Animal Care Policies and Guidelines for animal experiments.

### 2.2 Phylogenetic analyses

DNA barcodes of 657 basepairs of COI were analyzed from 346 specimens of *Molossus* from across the Neotropics including Bonaire, Dominican Republic, Ecuador, El Salvador, French Guiana, Guyana, Jamaica, Martinique, Mexico, Panama, Peru, Suriname, and Venezuela. The genera *Eumops* and *Promops* were included as outgroups following [7], [8]. Of these specimens, 50 are new sequences and 296 were obtained from GenBank (Supplementary material 1). Based on genetic divergence in CO1, a subset of 35 specimens spanning the breadth of variation was sequenced for 764 basepairs of the nuclear gene beta fibrinogen. Sequences were assembled in SEQUENCHER and aligned using the Muscle algorithm [5] as implemented in MEGA 6.06 [6]. Phylogenetic relationships were reconstructed for each single dataset using maximum likelihood analyses with 1000 bootstrap replications as implemented in MEGA 6.06 [6] (Figs. 1 and 2). Aligned datasets were subjected to alternative models of sequence evolution in Partition Finder 1.0.1 [9] to select the best partitions and models of sequence evolution [10].
