# Supplementary material for: Molecular data on the CO1 and beta fibrinogen gene in the evolutionary relationships of the mastiff bat (Chiroptera, Molossidae, Molossus)
Source: Data Brief. 2018 Apr 30;18:1609–13. doi: 10.1016/j.dib.2018.04.088 (PMC5998218; doi:10.1016/j.dib.2018.04.088)
Supplement: Supplementary file 1 — Supplementary material [file mmc1.pdf]

Declarations of interest:

none
