# Supplementary material for: Molecular data on the CO1 and beta fibrinogen gene in the evolutionary relationships of the mastiff bat (Chiroptera, Molossidae, Molossus)
Source: Data Brief. 2018 Apr 30;18:1609–13. doi: 10.1016/j.dib.2018.04.088 (PMC5998218; doi:10.1016/j.dib.2018.04.088)
Supplement: Supplementary file 2 — Supplementary material [file mmc2.docx]

**Supplementary material 1**

Specimen vouchers of *Molossus* used in the genetic analyses for the genes COI and beta fibrinogen, GenBank and BOLD Systems accessions numbers, species identification, and country.

| **Gene** | **Specimen #** | **GenBank** | **BOLD** | **Species** | **Country** |
| --- | --- | --- | --- | --- | --- |
| COI | ROM 115644 | EF080462 | BCBNT943-06 | *Molossus molossus* | Guyana |
| COI | ROM 115674 | EF080463 | BCBNT948-06 | *Molossus molossus* | Guyana |
| COI | ROM 115689 | EF080464 | BCBNT951-06 | *Molossus molossus* | Guyana |
| COI | ROM 115690 | EF080465 | BCBNT952-06 | *Molossus molossus* | Guyana |
| COI | ROM 115724 | EF080466 | BCBNT958-06 | *Molossus molossus* | Guyana |
| COI | ROM 115725 | EF080467 | BCBNT959-06 | *Molossus molossus* | Guyana |
| COI | ROM 115726 | EF080468 | BCBNT960-06 | *Molossus molossus* | Guyana |
| COI | ROM 115727 | EF080469 | BCBNT961-06 | *Molossus molossus* | Guyana |
| COI | ROM 115728 | EF080470 | BCBNT962-06 | *Molossus molossus* | Guyana |
| COI | ROM 112546 | EF080471 | BCBNT555-06 | *Molossus molossus* | Guyana |
| COI | ROM 115673 | EF080472 | BCBNT947-06 | *Molossus molossus* | Guyana |
| COI | ROM 109044 | EF080473 | BCBNT376-06 | *Molossus molossus* | Guyana |
| COI | ROM 109041 | EF080474 | BCBNT373-06 | *Molossus molossus* | Guyana |
| COI | ROM 109042 | EF080475 | BCBNT374-06 | *Molossus molossus* | Guyana |
| COI | ROM 109043 | EF080476 | BCBNT375-06 | *Molossus molossus* | Guyana |
| COI | ROM 109045 | EF080477 | BCBNT377-06 | *Molossus molossus* | Guyana |
| COI | ROM 107167 | EF080478 | BCBNT105-06 | *Molossus rufus* | Guyana |
| COI | ROM 107177 | EF080479 | BCBNT107-06 | *Molossus rufus* | Guyana |
| COI | ROM 108419 | EF080480 | BCBNT271-06 | *Molossus rufus* | Guyana |
| COI | ROM 108420 | EF080481 | BCBNT272-06 | *Molossus rufus* | Guyana |
| COI | ROM 108478 | EF080482 | BCBNT285-06 | *Molossus rufus* | Guyana |
| COI | ROM 109176 | EF080483 | BCBNT388-06 | *Molossus fentoni* sp. nov. | Guyana |
| COI | ROM 117682 | EU096788 | ABSMS520-06 | *Molossus molossus* | Suriname |
| COI | ROM 117659 | EU096789 | ABSMS497-06 | *Molossus molossus* | Suriname |
| COI | ROM 117661 | EU096790 | ABSMS499-06 | *Molossus molossus* | Suriname |
| COI | ROM 117657 | EU096791 | ABSMS495-06 | *Molossus molossus* | Suriname |
| COI | ROM 117660 | EU096792 | ABSMS498-06 | *Molossus molossus* | Suriname |
| COI | ROM 117656 | EU096793 | ABSMS494-06 | *Molossus molossus* | Suriname |
| COI | ROM 117658 | EU096794 | ABSMS496-06 | *Molossus molossus* | Suriname |
| COI | ROM 117550 | EU096795 | ABSMS388-06 | *Molossus rufus* | Suriname |
| COI | ROM 117466 | EU096796 | ABSMS304-06 | *Molossus rufus* | Suriname |
| COI | ROM 117477 | EU096797 | ABSMS315-06 | *Molossus rufus* | Suriname |
| COI | ROM 117549 | EU096798 | ABSMS387-06 | *Molossus rufus* | Suriname |
| COI | ROM 117476 | EU096799 | ABSMS314-06 | *Molossus rufus* | Suriname |
| COI | ROM 117475 | EU096800 | ABSMS313-06 | *Molossus rufus* | Suriname |
| COI | ROM 117474 | EU096801 | ABSMS312-06 | *Molossus rufus* | Suriname |
| COI | ROM 117473 | EU096802 | ABSMS311-06 | *Molossus rufus* | Suriname |
| COI | ROM 117479 | EU096803 | ABSMS317-06 | *Molossus rufus* | Suriname |
| COI | ROM 117478 | EU096804 | ABSMS316-06 | *Molossus rufus* | Suriname |
| COI | ROM 117481 | EU096805 | ABSMS319-06 | *Molossus rufus* | Suriname |
| COI | ROM 117548 | EU096806 | ABSMS386-06 | *Molossus rufus* | Suriname |
| COI | AVB080326-3 | HM208663 | ABVSC028-08 | *Molossus rufus* | Mexico |
| COI | T-4932 | HQ918468 | ABFG470-10 | *Molossus molossus* | Martinique |
| COI | ROM 120398 | HQ919749 | ABSRA1164-10 | *Molossus molossus* | Suriname |
| COI | ROM 120409 | HQ919759 | ABSRA1175-10 | *Molossus molossus* | Suriname |
| COI | ROM 120410 | HQ919760 | ABSRA1176-10 | *Molossus molossus* | Suriname |
| COI | ROM 120411 | HQ919761 | ABSRA1177-10 | *Molossus molossus* | Suriname |
| COI | ROM 120412 | HQ919762 | ABSRA1178-10 | *Molossus molossus* | Suriname |
| COI | ROM 120413 | HQ919763 | ABSRA1179-10 | *Molossus molossus* | Suriname |
| COI | ASK7655 | JF442198 | GBMA4826-13 | *Molossus molossus* | Ecuador |
| COI | ASK7663 | JF442199 | GBMA4825-13 | *Molossus molossus* | Ecuador |
| COI | ASK7664 | JF442200 | GBMA4824-13 | *Molossus molossus* | Ecuador |
| COI | ASK7691 | JF442201 | GBMA4823-13 | *Molossus coibensis* | Ecuador |
| COI | ASK7695 | JF442202 | GBMA4822-13 | *Molossus molossus* | Ecuador |
| COI | ASK7696 | JF442203 | GBMA4821-13 | *Molossus molossus* | Ecuador |
| COI | ASK7697 | JF442204 | GBMA4820-13 | *Molossus molossus* | Ecuador |
| COI | ASK7713 | JF442205 | GBMA4819-13 | *Molossus molossus* | Ecuador |
| COI | ASK7720 | JF442206 | GBMA4818-13 | *Molossus molossus* | Ecuador |
| COI | ASK7721 | JF442207 | GBMA4817-13 | *Molossus molossus* | Ecuador |
| COI | ASK7722 | JF442208 | GBMA4816-13 | *Molossus molossus* | Ecuador |
| COI | ASK7727 | JF442209 | GBMA4815-13 | *Molossus molossus* | Ecuador |
| COI | ASK7728 | JF442210 | GBMA4814-13 | *Molossus molossus* | Ecuador |
| COI | ASK7729 | JF442211 | GBMA4813-13 | *Molossus molossus* | Ecuador |
| COI | ASK7730 | JF442212 | GBMA4812-13 | *Molossus molossus* | Ecuador |
| COI | ASK7731 | JF442213 | GBMA4811-13 | *Molossus molossus* | Ecuador |
| COI | ASK7732 | JF442214 | GBMA4810-13 | *Molossus molossus* | Ecuador |
| COI | ASK7739 | JF442215 | GBMA4809-13 | *Molossus molossus* | Ecuador |
| COI | ASK7740 | JF442216 | GBMA4808-13 | *Molossus molossus* | Ecuador |
| COI | ASK7741 | JF442217 | GBMA4807-13 | *Molossus molossus* | Ecuador |
| COI | ASK7742 | JF442218 | GBMA4806-13 | *Molossus molossus* | Ecuador |
| COI | ASK7744 | JF442220 | GBMA4804-13 | *Molossus molossus* | Ecuador |
| COI | ASK7745 | JF442221 | GBMA4803-13 | *Molossus molossus* | Ecuador |
| COI | ASK7747 | JF442222 | GBMA4802-13 | *Molossus molossus* | Ecuador |
| COI | ASK7749 | JF442224 | GBMA4800-13 | *Molossus molossus* | Ecuador |
| COI | ASK7753 | JF442226 | GBMA4798-13 | *Molossus rufus* | Ecuador |
| COI | ASK7756 | JF442227 | GBMA4797-13 | *Molossus molossus* | Ecuador |
| COI | ASK7757 | JF442228 | GBMA4796-13 | *Molossus molossus* | Ecuador |
| COI | ASK7759 | JF442229 | GBMA4795-13 | *Molossus molossus* | Ecuador |
| COI | ASK7760 | JF442230 | GBMA4794-13 | *Molossus molossus* | Ecuador |
| COI | ASK7761 | JF442231 | GBMA4793-13 | *Molossus molossus* | Ecuador |
| COI | ASK7762 | JF442232 | GBMA4792-13 | *Molossus molossus* | Ecuador |
| COI | ASK7763 | JF442233 | GBMA4791-13 | *Molossus molossus* | Ecuador |
| COI | ASK7764 | JF442234 | GBMA4790-13 | *Molossus molossus* | Ecuador |
| COI | ASK7774 | JF442236 | GBMA4788-13 | *Molossus m. daulensis* | Ecuador |
| COI | ASK7777 | JF442237 | GBMA4787-13 | *Molossus m. daulensis* | Ecuador |
| COI | ASK7778 | JF442238 | GBMA4786-13 | *Molossus m. daulensis* | Ecuador |
| COI | ASK7779 | JF442239 | GBMA4785-13 | *Molossus m. daulensis* | Ecuador |
| COI | ASK7785 | JF442240 | GBMA4784-13 | *Molossus m. daulensis* | Ecuador |
| COI | ASK7786 | JF442241 | GBMA4783-13 | *Molossus m. daulensis* | Ecuador |
| COI | ASK7787 | JF442242 | GBMA4782-13 | *Molossus m. daulensis* | Ecuador |
| COI | ASK7788 | JF442243 | GBMA4781-13 | *Molossus m. daulensis* | Ecuador |
| COI | ASK7789 | JF442244 | GBMA4780-13 | *Molossus m. daulensis* | Ecuador |
| COI | ASK7790 | JF442245 | GBMA4779-13 | *Molossus m. daulensis* | Ecuador |
| COI | ROM 118784 | JF444936 | ABECB122-08 | *Promops centralis* | Ecuador |
| COI | ROM 106035 | JF444940 | ABECA859-06 | *Promops centralis* | Ecuador |
| COI | ROM 101279 | JF446514 | BCBN395-05 | *Molossus rufus* | El Salvador |
| COI | ROM 101278 | JF446515 | ABCSA715-06 | *Molossus rufus* | El Salvador |
| COI | ROM 101287 | JF446516 | ABCSA719-06 | *Molossus rufus* | El Salvador |
| COI | ROM 101285 | JF446517 | ABCSA718-06 | *Molossus rufus* | El Salvador |
| COI | ROM 101283 | JF446518 | ABCSA717-06 | *Molossus rufus* | El Salvador |
| COI | ROM 101282 | JF446519 | ABCSA716-06 | *Molossus rufus* | El Salvador |
| COI | ROM 96186 | JF447269 | BCBN020-05 | *Molossus rufus* | Mexico |
| COI | ROM 104194 | JF447423 | BCBN601-05 | *Molossus molossus* | Panama |
| COI | ROM 113902 | JF447680 | BCBNT731-06 | *Molossus molossus* | Suriname |
| COI | ROM 113900 | JF447681 | BCBNT729-06 | *Molossus molossus* | Suriname |
| COI | ROM 113950 | JF447682 | BCBNT755-06 | *Molossus molossus* | Suriname |
| COI | ROM 113899 | JF447683 | BCBNT728-06 | *Molossus molossus* | Suriname |
| COI | ROM 113901 | JF447684 | BCBNT730-06 | *Molossus molossus* | Suriname |
| COI | ROM 113903 | JF447685 | BCBNT732-06 | *Molossus rufus* | Suriname |
| COI | ROM 117007 | JF447686 | ABSRA418-06 | *Molossus rufus* | Suriname |
| COI | ROM 117000 | JF447687 | ABSRA411-06 | *Molossus rufus* | Suriname |
| COI | ROM 116999 | JF447688 | ABSRA410-06 | *Molossus rufus* | Suriname |
| COI | ROM 107901 | JF447832 | BCBNT196-06 | *Molossus sp.* | Venezuela |
| COI | ROM 107900 | JF447833 | BCBNT195-06 | *Molossus sp.* | Venezuela |
| COI | ROM 107869 | JF447834 | BCBNT181-06 | *Molossus sp.* | Venezuela |
| COI | ROM 105638 | JF448088 | BCBN862-05 | *Molossus coibensis* | Ecuador |
| COI | ROM 101286 | JF448089 | BCBN399-05 | *Molossus rufus* | El Salvador |
| COI | ROM 101280 | JF448090 | BCBN396-05 | *Molossus rufus* | El Salvador |
| COI | ROM 101284 | JF448091 | BCBN398-05 | *Molossus rufus* | El Salvador |
| COI | ROM 101281 | JF448092 | BCBN397-05 | *Molossus rufus* | El Salvador |
| COI | ROM 105602 | JF448093 | BCBN855-05 | *Molossus rufus* | Ecuador |
| COI | ROM 105688 | JF448946 | BCBN880-05 | *Molossus coibensis* | Ecuador |
| COI | ROM 105303 | JF448947 | BCBN819-05 | *Molossus coibensis* | Ecuador |
| COI | ROM 105687 | JF448948 | BCBN879-05 | *Molossus coibensis* | Ecuador |
| COI | ROM 105690 | JF448949 | BCBN881-05 | *Molossus coibensis* | Ecuador |
| COI | ROM 105637 | JF448950 | BCBN861-05 | *Molossus coibensis* | Ecuador |
| COI | ROM F41830 | JF448951 | ABRMM098-07 | *Molossus coibensis* | Ecuador |
| COI | ROM 105590 | JF448952 | ABECA561-06 | *Molossus molossus* | Ecuador |
| COI | ROM 105689 | JF448953 | ABECA637-06 | *Molossus molossus* | Ecuador |
| COI | ROM 105568 | JF448954 | ABECA541-06 | *Molossus molossus* | Ecuador |
| COI | ROM 105542 | JF448955 | ABECA507-06 | *Molossus molossus* | Ecuador |
| COI | ROM 105569 | JF448956 | ABECA542-06 | *Molossus molossus* | Ecuador |
| COI | ROM 105543 | JF448957 | ABECA508-06 | *Molossus molossus* | Ecuador |
| COI | ROM 105544 | JF448958 | ABECA509-06 | *Molossus molossus* | Ecuador |
| COI | ROM 105545 | JF448959 | ABECA510-06 | *Molossus molossus* | Ecuador |
| COI | ROM 104435 | JF448960 | ABECA137-06 | *Molossus molossus* | Ecuador |
| COI | ROM 104018 | JF448961 | ABECA036-06 | *Molossus molossus* | Ecuador |
| COI | ROM 105367 | JF448962 | ABECA457-06 | *Molossus molossus* | Ecuador |
| COI | ROM 104022 | JF448963 | ABECA040-06 | *Molossus molossus* | Ecuador |
| COI | ROM 105360 | JF448964 | ABECA450-06 | *Molossus molossus* | Ecuador |
| COI | ROM 105514 | JF448965 | ABECA491-06 | *Molossus molossus* | Ecuador |
| COI | ROM 105878 | JF448966 | BCBN944-05 | *Molossus molossus* | Ecuador |
| COI | ROM 105926 | JF448967 | BCBN954-05 | *Molossus molossus* | Ecuador |
| COI | ROM 105356 | JF448968 | BCBN828-05 | *Molossus molossus* | Ecuador |
| COI | ROM 106054 | JF448969 | BCBN978-05 | *Molossus molossus* | Ecuador |
| COI | ROM 106053 | JF448970 | BCBN977-05 | *Molossus molossus* | Ecuador |
| COI | ROM 106324 | JF448971 | ABECB007-06 | *Molossus molossus* | Ecuador |
| COI | ROM 118785 | JF448972 | ABECB123-08 | *Molossus molossus* | Ecuador |
| COI | ROM 106325 | JF448973 | ABECB008-06 | *Molossus molossus* | Ecuador |
| COI | ROM F40478 | JF448974 | ABECB006-06 | *Molossus molossus* | Ecuador |
| COI | ROM F41838 | JF448975 | ABRMM106-07 | *Molossus molossus* | Ecuador |
| COI | ROM F41837 | JF448976 | ABRMM105-07 | *Molossus molossus* | Ecuador |
| COI | ROM F41836 | JF448977 | ABRMM104-07 | *Molossus molossus* | Ecuador |
| COI | ROM 105355 | JF448978 | BCBN827-05 | *Molossus rufus* | Ecuador |
| COI | ROM 105686 | JF448979 | BCBN878-05 | *Molossus rufus* | Ecuador |
| COI | ROM 105681 | JF448980 | BCBN875-05 | *Molossus rufus* | Ecuador |
| COI | ROM 105682 | JF448981 | ABECA635-06 | *Molossus rufus* | Ecuador |
| COI | ROM 105304 | JF448982 | ABECA405-06 | *Molossus rufus* | Ecuador |
| COI | ROM F41833 | JF448983 | ABRMM101-07 | *Molossus rufus* | Ecuador |
| COI | ROM 106020 | JF449067 | BCBN973-05 | *Promops centralis* | Ecuador |
| COI | ROM F41839 | JF449068 | ABRMM107-07 | *Promops centralis* | Ecuador |
| COI | ROM F41835 | JF449069 | ABRMM103-07 | *Promops centralis* | Ecuador |
| COI | ROM 111992 | JF455012 | ABGYE520-06 | *Molossus molossus* | Guyana |
| COI | ROM 109046 | JF455013 | ABGYD638-06 | *Molossus molossus* | Guyana |
| COI | ROM 116525 | JF455014 | ABGYG397-06 | *Molossus molossus* | Guyana |
| COI | ROM 113813 | JF455015 | ABGYF235-06 | *Molossus molossus* | Guyana |
| COI | ROM 113750 | JF455016 | ABGYF189-06 | *Molossus molossus* | Guyana |
| COI | ROM 113814 | JF455017 | ABGYF236-06 | *Molossus molossus* | Guyana |
| COI | ROM 111993 | JF455018 | ABGYE521-06 | *Molossus molossus* | Guyana |
| COI | ROM 111980 | JF455019 | ABGYE508-06 | *Molossus molossus* | Guyana |
| COI | ROM 116634 | JF455020 | ABGYG493-06 | *Molossus molossus* | Guyana |
| COI | ROM 119128 | JF455021 | ABGYG700-08 | *Molossus molossus* | Guyana |
| COI | ROM 116652 | JF455022 | ABGYG510-06 | *Molossus molossus* | Guyana |
| COI | ROM F39787 | JF455023 | ABGYC730-06 | *Molossus molossus* | Guyana |
| COI | ROM 112700 | JF455024 | ABGYE754-06 | *Molossus molossus* | Guyana |
| COI | ROM 111991 | JF455025 | ABGYE519-06 | *Molossus molossus* | Guyana |
| COI | ROM 111990 | JF455026 | ABGYE518-06 | *Molossus molossus* | Guyana |
| COI | ROM 111989 | JF455027 | ABGYE517-06 | *Molossus molossus* | Guyana |
| COI | ROM 112688 | JF455028 | ABGYE745-06 | *Molossus molossus* | Guyana |
| COI | ROM 111988 | JF455029 | ABGYE516-06 | *Molossus molossus* | Guyana |
| COI | ROM 111987 | JF455030 | ABGYE515-06 | *Molossus molossus* | Guyana |
| COI | ROM 111986 | JF455031 | ABGYE514-06 | *Molossus molossus* | Guyana |
| COI | ROM 111985 | JF455032 | ABGYE513-06 | *Molossus molossus* | Guyana |
| COI | ROM 112689 | JF455033 | ABGYE746-06 | *Molossus molossus* | Guyana |
| COI | ROM 112035 | JF455034 | ABGYE562-06 | *Molossus molossus* | Guyana |
| COI | ROM 109049 | JF455035 | ABGYD641-06 | *Molossus molossus* | Guyana |
| COI | ROM 109048 | JF455036 | ABGYD640-06 | *Molossus molossus* | Guyana |
| COI | ROM 109060 | JF455037 | ABGYD652-06 | *Molossus molossus* | Guyana |
| COI | ROM 109059 | JF455038 | ABGYD651-06 | *Molossus molossus* | Guyana |
| COI | ROM 109058 | JF455039 | ABGYD650-06 | *Molossus molossus* | Guyana |
| COI | ROM 109057 | JF455040 | ABGYD649-06 | *Molossus molossus* | Guyana |
| COI | ROM 109056 | JF455041 | ABGYD648-06 | *Molossus molossus* | Guyana |
| COI | ROM 109055 | JF455042 | ABGYD647-06 | *Molossus molossus* | Guyana |
| COI | ROM 109054 | JF455043 | ABGYD646-06 | *Molossus molossus* | Guyana |
| COI | ROM 109053 | JF455044 | ABGYD645-06 | *Molossus molossus* | Guyana |
| COI | ROM 109052 | JF455045 | ABGYD644-06 | *Molossus molossus* | Guyana |
| COI | ROM 109051 | JF455046 | ABGYD643-06 | *Molossus molossus* | Guyana |
| COI | ROM 109047 | JF455047 | ABGYD639-06 | *Molossus molossus* | Guyana |
| COI | ROM 109050 | JF455048 | ABGYD642-06 | *Molossus molossus* | Guyana |
| COI | ROM 106554 | JF455049 | ABGYC067-06 | *Molossus molossus* | Guyana |
| COI | ROM 108126 | JF455050 | ABGYC726-06 | *Molossus molossus* | Guyana |
| COI | ROM 108127 | JF455051 | ABGYC727-06 | *Molossus molossus* | Guyana |
| COI | ROM F39786 | JF455052 | ABGYC729-06 | *Molossus molossus* | Guyana |
| COI | ROM 97773 | JF455053 | ABGYA005-06 | *Molossus molossus* | Guyana |
| COI | ROM 98716 | JF455054 | ABGYA318-06 | *Molossus molossus* | Guyana |
| COI | ROM 97775 | JF455055 | ABGYA007-06 | *Molossus molossus* | Guyana |
| COI | ROM 98717 | JF455056 | ABGYA319-06 | *Molossus molossus* | Guyana |
| COI | ROM 98718 | JF455057 | ABGYA320-06 | *Molossus molossus* | Guyana |
| COI | ROM 98720 | JF455058 | ABGYA322-06 | *Molossus molossus* | Guyana |
| COI | ROM 98721 | JF455059 | ABGYA323-06 | *Molossus molossus* | Guyana |
| COI | ROM 98722 | JF455060 | ABGYA324-06 | *Molossus molossus* | Guyana |
| COI | ROM 97802 | JF455061 | ABGYA020-06 | *Molossus molossus* | Guyana |
| COI | ROM 97803 | JF455062 | ABGYA021-06 | *Molossus molossus* | Guyana |
| COI | ROM 97804 | JF455063 | ABGYA022-06 | *Molossus molossus* | Guyana |
| COI | ROM 97805 | JF455064 | ABGYA023-06 | *Molossus molossus* | Guyana |
| COI | ROM 97806 | JF455065 | ABGYA024-06 | *Molossus molossus* | Guyana |
| COI | ROM 97807 | JF455066 | ABGYA025-06 | *Molossus molossus* | Guyana |
| COI | ROM 97808 | JF455067 | ABGYA026-06 | *Molossus molossus* | Guyana |
| COI | ROM 97809 | JF455068 | ABGYA027-06 | *Molossus molossus* | Guyana |
| COI | ROM 98703 | JF455069 | ABGYA307-06 | *Molossus molossus* | Guyana |
| COI | ROM 103559 | JF455070 | ABGYB791-06 | *Molossus molossus* | Guyana |
| COI | ROM 103560 | JF455071 | ABGYB792-06 | *Molossus molossus* | Guyana |
| COI | ROM 103561 | JF455072 | ABGYB793-06 | *Molossus molossus* | Guyana |
| COI | ROM 103562 | JF455073 | ABGYB794-06 | *Molossus molossus* | Guyana |
| COI | ROM 103563 | JF455074 | ABGYB795-06 | *Molossus molossus* | Guyana |
| COI | ROM 103564 | JF455075 | ABGYB796-06 | *Molossus molossus* | Guyana |
| COI | ROM 103565 | JF455076 | ABGYB797-06 | *Molossus molossus* | Guyana |
| COI | ROM 103566 | JF455077 | ABGYB798-06 | *Molossus molossus* | Guyana |
| COI | ROM 103567 | JF455078 | ABGYB799-06 | *Molossus molossus* | Guyana |
| COI | ROM 103568 | JF455079 | ABGYB800-06 | *Molossus molossus* | Guyana |
| COI | ROM 103569 | JF455080 | ABGYB801-06 | *Molossus molossus* | Guyana |
| COI | ROM 103556 | JF455081 | ABGYB788-06 | *Molossus molossus* | Guyana |
| COI | ROM 103570 | JF455082 | ABGYB802-06 | *Molossus molossus* | Guyana |
| COI | ROM 103571 | JF455083 | ABGYB803-06 | *Molossus molossus* | Guyana |
| COI | ROM 103572 | JF455084 | ABGYB804-06 | *Molossus molossus* | Guyana |
| COI | ROM 103573 | JF455085 | ABGYB805-06 | *Molossus molossus* | Guyana |
| COI | ROM 103574 | JF455086 | ABGYB806-06 | *Molossus molossus* | Guyana |
| COI | ROM 103575 | JF455087 | ABGYB807-06 | *Molossus molossus* | Guyana |
| COI | ROM 103576 | JF455088 | ABGYB808-06 | *Molossus molossus* | Guyana |
| COI | ROM 103557 | JF455089 | ABGYB789-06 | *Molossus molossus* | Guyana |
| COI | ROM 103577 | JF455090 | ABGYB809-06 | *Molossus molossus* | Guyana |
| COI | ROM 103558 | JF455091 | ABGYB790-06 | *Molossus molossus* | Guyana |
| COI | ROM F39071 | JF455092 | ABRMM064-07 | *Molossus rufus* | Guyana |
| COI | ROM 108422 | JF455093 | ABGYD122-06 | *Molossus rufus* | Guyana |
| COI | ROM 108421 | JF455094 | ABGYD121-06 | *Molossus rufus* | Guyana |
| COI | ROM 109177 | JF455095 | ABGYD758-06 | *Molossus rufus* | Guyana |
| COI | ROM 108480 | JF455096 | ABGYD166-06 | *Molossus rufus* | Guyana |
| COI | ROM 108479 | JF455097 | ABGYD165-06 | *Molossus rufus* | Guyana |
| COI | ROM 109154 | JF455098 | ABGYD737-06 | *Molossus rufus* | Guyana |
| COI | ROM F39068 | JF455099 | ABGYC611-06 | *Molossus rufus* | Guyana |
| COI | ROM 107250 | JF455100 | ABGYC428-06 | *Molossus rufus* | Guyana |
| COI | ROM 119877 | JF459205 | ABGYG1230-08 | *Molossus molossus* | Guyana |
| COI | ROM 119842 | JF459206 | ABGYG1195-08 | *Molossus molossus* | Guyana |
| COI | ROM 119841 | JF459207 | ABGYG1194-08 | *Molossus molossus* | Guyana |
| COI | ROM 119809 | JF459208 | ABGYG1162-08 | *Molossus molossus* | Guyana |
| COI | ROM 119808 | JF459209 | ABGYG1161-08 | *Molossus molossus* | Guyana |
| COI | ROM 119807 | JF459210 | ABGYG1160-08 | *Molossus molossus* | Guyana |
| COI | ROM 119806 | JF459211 | ABGYG1159-08 | *Molossus molossus* | Guyana |
| COI | ROM 119804 | JF459212 | ABGYG1157-08 | *Molossus molossus* | Guyana |
| COI | ROM 119803 | JF459213 | ABGYG1156-08 | *Molossus molossus* | Guyana |
| COI | ROM 119833 | JF459214 | ABGYG1186-08 | *Molossus rufus* | Guyana |
| COI | ROM 119707 | JQ601428 | ABSRA760-08 | *Molossus molossus* | Suriname |
| COI | ROM 119708 | JQ601429 | ABSRA761-08 | *Molossus molossus* | Suriname |
| COI | T-4602 |  | ABFG423-10 | *Molossus coibensis* | French Guiana |
| COI | ROM 108128 |  | ABGYC728-06 | *Molossus molossus* | Guyana |
| COI | ROM 107256 |  | ABGYC432-06 | *Molossus molossus* | Guyana |
| COI | ROM 119706 |  | ABGYH117-12 | *Molossus molossus* | Suriname |
| COI | ROM 119709 |  | ABGYH118-12 | *Molossus molossus* | Suriname |
| COI | ROM F41841 |  | ABGYH150-12 | *Promops centralis* | Ecuador |
| COI | ROM F41834 |  | ABGYH148-12 | *Promops centralis* | Ecuador |
| COI | ROM 118821 | | ABRMM100-07 | *Molossus fentoni sp. nov.* | Ecuador |
| COI | ROM 120414 | | ABSRA1180-10 | *Molossus molossus* | Suriname |
| COI | ROM F41831 | | ABRMM099-07 | *Molossus coibensis* | Ecuador |
| COI | ROM 101097 | | BCBN360-05 | *Molossus coibensis* | Guyana |
| COI | ROM 105927 | | BCBN955-05 | *Molossus rufus* | Ecuador |
| COI | ROM 119012 | | ABGYG584-08 | *Molossus sp.* | Guyana |
| COI | ROM 120796 | KT023205 | | *Molossus m. milleri* | Jamaica |
| COI | ROM 120797 | KT023206 | | *Molossus m. milleri* | Jamaica |
| COI | ROM 120798 | KT023207 | | *Molossus m. milleri* | Jamaica |
| COI | ROM 120799 | KT023208 | | *Molossus m. milleri* | Jamaica |
| COI | ROM 120818 | KT023209 | | *Molossus m. milleri* | Jamaica |
| COI | ROM 120819 | KT023210 | | *Molossus m. milleri* | Jamaica |
| COI | ROM 120820 | KT023211 | | *Molossus m. milleri* | Jamaica |
| COI | ROM 120847 | KT023212 | | *Molossus m. milleri* | Jamaica |
| COI | ROM 120848 | KT023213 | | *Molossus m. milleri* | Jamaica |
| COI | ROM 120849 | KT023214 | | *Molossus m. milleri* | Jamaica |
| COI | ROM 120850 | KT023215 | | *Molossus m. milleri* | Jamaica |
| COI | ROM 125286 | KX355059 | | *Molossus m. verrilli* | Dominican Republic |
| COI | ROM 125287 | KX355060 | | *Molossus m. verrilli* | Dominican Republic |
| COI | ROM 125288 | KX355061 | | *Molossus m. verrilli* | Dominican Republic |
| COI | ROM 125289 | KX355062 | | *Molossus m. verrilli* | Dominican Republic |
| COI | ROM 125385 | KX355063 | | *Molossus m. verrilli* | Dominican Republic |
| COI | ROM 125386 | KX355064 | | *Molossus m. verrilli* | Dominican Republic |
| COI | ROM 125387 | KX355065 | | *Molossus m. verrilli* | Dominican Republic |
| COI | ROM 125388 | KX355066 | | *Molossus m. verrilli* | Dominican Republic |
| COI | TTU104174 | KX959997 | | *Molossus sinaloae* | Honduras |
| COI | AMNH269107 | KX959998 | | *Molossus sinaloae* | French Guiana |
| COI | AMNH267549 | KX959999 | | *Molossus sinaloae* | French Guiana |
| COI | AMNH269110 | KX960000 | | *Molossus sinaloae* | French Guiana |
| COI | ROM 125541 | KX960001 | | *Molossus molossus* | Peru |
| COI | ROM 125542 | KX960002 | | *Molossus molossus* | Peru |
| COI | ROM 125543 | KX960003 | | *Molossus molossus* | Peru |
| COI | ROM 125544 | KX960004 | | *Molossus molossus* | Peru |
| COI | ROM 125553 | KX960005 | | *Molossus rufus* | Peru |
| COI | ROM 125554 | KX960006 | | *Molossus molossus* | Peru |
| COI | ROM 125571 | KX960007 | | *Molossus molossus* | Peru |
| COI | ROM 125597 | KX960008 | | *Molossus molossus* | Peru |
| COI | ROM 125598 | KX960009 | | *Molossus molossus* | Peru |
| COI | ROM 125468 | KX960010 | | *Molossus molossus* | Bonaire |
| COI | ROM 125274 | KX960011 | | *Molossus molossus* | Guyana |
| COI | ROM 125275 | KX960012 | | *Molossus molossus* | Guyana |
| COI | ROM 125233 | KX960013 | | *Molossus rufus* | Guyana |
| COI | ROM 125255 | KX960014 | | *Molossus rufus* | Guyana |
| COI | ROM 125272 | KX960015 | | *Molossus rufus* | Guyana |
| COI | ROM 125273 | KX960016 | | *Molossus rufus* | Guyana |
| COI | ROM 120415 | KX960017 | | *Molossus molossus* | Suriname |
| COI | ROM 122087 | KX960018 | | *Molossus coibensis* | Peru |
| COI | ROM 122091 | KX960019 | | *Molossus coibensis* | Peru |
| COI | ROM 122130 | KX960020 | | *Molossus coibensis* | Peru |
| COI | ROM 122177 | KX960021 | | *Molossus coibensis* | Peru |
| COI | ROM 122254 | KX960022 | | *Molossus coibensis* | Peru |
| COI | ROM 122255 | KX960023 | | *Molossus coibensis* | Peru |
| COI | ROM 122119 | KX960024 | | *Molossus molossus* | Peru |
| COI | ROM 122146 | KX960025 | | *Molossus molossus* | Peru |
| COI | ROM 122147 | KX960026 | | *Molossus molossus* | Peru |
| COI | ROM 122148 | KX960027 | | *Molossus molossus* | Peru |
| COI | ROM 122152 | KX960028 | | *Molossus molossus* | Peru |
| COI | ROM 122253 | KX960029 | | *Molossus molossus* | Peru |
| COI | ROM 122256 | KX960030 | | *Molossus molossus* | Peru |
| COI | ROM 122090 | KX960031 | | *Molossus rufus* | Peru |
| COI | ROM 122116 | KX960032 | | *Molossus rufus* | Peru |
| COI | ROM 121974 | KX960033 | | *Molossus molossus* | Guyana |
| COI | ROM 122010 | KX960034 | | *Molossus molossus* | Guyana |
| COI | ROM 122401 | KX960035 | | *Molossus molossus* | Guyana |
| COI | ROM 122422 | KX960036 | | *Molossus molossus* | Guyana |
| COI | ROM 122427 | KX960037 | | *Molossus molossus* | Guyana |
| COI | ROM 122433 | KX960038 | | *Molossus molossus* | Guyana |
| COI | ROM 122441 | KX960039 | | *Molossus molossus* | Guyana |
| COI | ROM 122608 | KX960040 | | *Molossus molossus* | Guyana |
| COI | ROM 122583 | KX960041 | | *Molossus* *fentoni sp. nov.* | Guyana |
| COI | ROM 125074 | KX960042 | | *Molossus molossus* | Guyana |
| COI | ROM 125075 | KX960043 | | *Molossus molossus* | Guyana |
| COI | ROM 121129 | KX960044 | | *Molossus rufus* | Suriname |
| Beta fibrinogen | AMNH 269107 | MF436707 | | *Molossus sinaloae* | French Guiana |
| Beta fibrinogen | ROM 107167 | MF436708 | | *Molossus rufus* | Guyana |
| Beta fibrinogen | ROM 121129 | MF436709 | | *Molossus rufus* | Suriname |
| Beta fibrinogen | ROM 122090 | MF436710 | | *Molossus rufus* | Peru |
| Beta fibrinogen | ROM 118784 | MF436711 | | *Molossus rufus* | Ecuador |
| Beta fibrinogen | ROM F39068 | MF436712 | | *Molossus rufus* | Guyana |
| Beta fibrinogen | ROM 101278 | MF436713 | | *Molossus rufus* | El Salvador |
| Beta fibrinogen | MSB 75535 | MF436714 | | *Molossus rufus* | Mexico |
| Beta fibrinogen | FMNH 174945 | MF436715 | | *Molossus rufus* | Peru |
| Beta fibrinogen | ROM 107901 | MF436716 | | *Molossus* sp. | Venezuela |
| Beta fibrinogen | ROM 107900 | MF436717 | | *Molossus* sp. | Venezuela |
| Beta fibrinogen | ROM 117660 | MF436718 | | *Molossus molossus* | Suriname |
| Beta fibrinogen | ROM 113899 | MF436719 | | *Molossus molossus* | Suriname |
| Beta fibrinogen | ROM 122254 | MF436720 | | *Molossus molossus* | Peru |
| Beta fibrinogen | ROM 122253 | MF436721 | | *Molossus molossus* | Peru |
| Beta fibrinogen | OMNH 7438 | MF436722 | | *Molossus molossus* | Peru |
| Beta fibrinogen | MZV Mamm 168936 | MF436723 | | *Molossus molossus* | Peru |
| Beta fibrinogen | MZV Mamm 166225 | MF436724 | | *Molossus molossus* | and |
| Beta fibrinogen | ROM 120821 | MF436725 | | *Molossus verrilli* | Jamaica |
| Beta fibrinogen | ROM 120796 | MF436726 | | *Molossus verrilli* | Jamaica |
| Beta fibrinogen | ROM 108128 | MF436727 | | *Molossus molossus* | Guyana |
| Beta fibrinogen | ROM 108127 | MF436728 | | *Molossus molossus* | Guyana |
| Beta fibrinogen | ROM 118785 | MF436729 | | *Molossus molossus* | Ecuador |
| Beta fibrinogen | ROM 104018 | MF436730 | | *Molossus molossus* | Ecuador |
| Beta fibrinogen | ROM 120848 | MF436731 | | *Molossus milleri* | Dominican Republic |
| Beta fibrinogen | ROM 125387 | MF436732 | | *Molossus milleri* | Dominican Republic |
| Beta fibrinogen | MSB 210952 | MF436733 | | *Molossus molossus* | Bolivia |
| Beta fibrinogen | ROM 122583 | MF436734 | | *Molossus* *fentoni sp. nov.* | Guyana |
| Beta fibrinogen | ROM 109176 | MF436735 | | *Molossus* *fentoni sp. nov.* | Guyana |
| Beta fibrinogen | ROM 122091 | MF436736 | | *Molossus coibensis* | Peru |
| Beta fibrinogen | ROM 122087 | MF436737 | | *Molossus coibensis* | Peru |
| Beta fibrinogen | ROM 122087 | MF436737 | | *Molossus coibensis* | Peru |
| Beta fibrinogen | ROM 105304 | MF436738 | | *Molossus rufus* | Ecuador |
| Beta fibrinogen | ROM 108918 | MF436739 | | *Eumops auripendulus* | Guyana |
| Beta fibrinogen | ROM 108917 | MF436740 | | *Eumops auripendulus* | Guyana |
| Beta fibrinogen | ROM 106035 | MF436741 | | *Promops centralis* | Ecuador |
